# Introgression Threatens the Survival of the Critically Endangered Freshwater Crayfish *Cherax tenuimanus* (Decapoda: Parastacidae) in the Wild

**DOI:** 10.1371/journal.pone.0121075

**Published:** 2015-03-23

**Authors:** Clodagh Guildea, Yvette Hitchen, Rodney Duffy, P. Joana Dias, Jason M. Ledger, Michael Snow, W. Jason Kennington

**Affiliations:** 1 Centre for Evolutionary Biology, School of Animal Biology, The University of Western Australia, Crawley, WA 6009, Australia; 2 Helix Molecular Solutions, PO Box 155, Leederville, WA 6903, Australia; 3 Department of Fisheries, Government of Western Australia, Western Australian Fisheries and Marine Research Laboratories, PO Box 20, North Beach, WA 6920, Australia; 4 School of Animal Biology, The University of Western Australia, Crawley, WA 6009, Australia; Bournemouth University, UNITED KINGDOM

## Abstract

Hybridization and genetic introgression following the introduction of exotic species can pose a significant threat to the survival of geographically restricted species. A remnant population of the critically endangered freshwater crayfish *Cherax tenuimanus* in the upper reaches of the Margaret River in southwestern Australia is under threat following the introduction and spread of its congener *Cherax cainii*. Here, we examine the extent of hybridization and introgression between the two species using twelve polymorphic microsatellite loci. Our study reveals there are three times more *C*. *cainii* than *C*. *tenuimanus* at our study site in the upper reaches of the Margaret River. There is also evidence of hybridization and introgression between *C*. *tenuimanus* and *C*. *cainii* at this site, with F_1_, F_2_ and backcrossed individuals identified. While interbreeding was confirmed in this study, our simulations suggest that the levels of introgression are much lower than would be expected under random mating, indicating partial reproductive barriers exist. Nevertheless, it is apparent that hybridization and introgression with *C*. *cainii* pose a serious threat to *C*. *tenuimanus* and their survival in the wild will require active adaptive management and continued genetic monitoring to evaluate management effectiveness.

## Introduction

The survival of a species can be detrimentally affected by invasive species, especially if accompanied by hybridization and introgression [1–3]. The threat of introgression is particularly apparent when rare species come into contact with more abundant relatives, as they are vulnerable to genetic swamping [1,4–7]. When introgression spreads throughout a species’ range, the species effectively becomes extinct and subsequent recovery is not possible. It therefore poses a significant threat to geographically restricted species [8].

**Fig 1:**
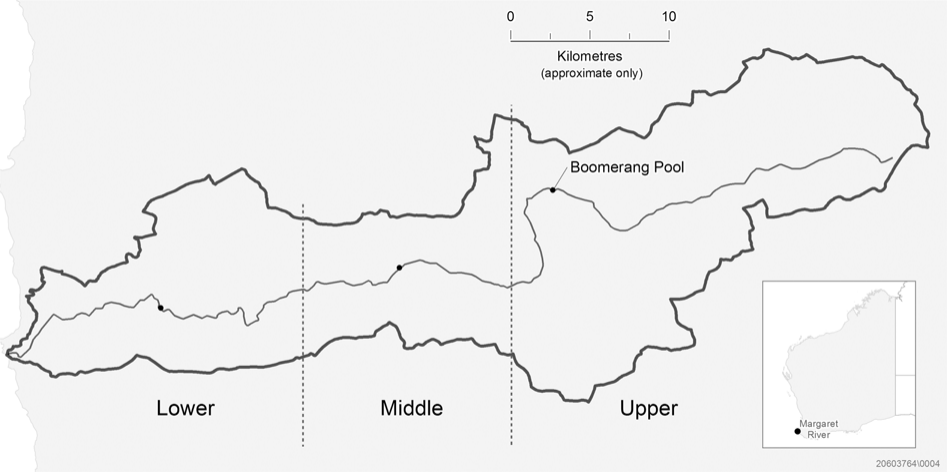
A map displaying the Margaret River system divided into its lower, middle and upper reaches. Boomerang Pool (marked) is the site where marron were collected for this study.

The hairy marron *Cherax tenuimanus* (Decapoda: Parastacidae) [9], is a freshwater crayfish endemic to the southwest of Western Australia. It has been listed as critically endangered under the Western Australian Wildlife Conservation Act 1950 and the IUCN Red List of Threatened Species. *Cherax tenuimanus* currently persists within the upper reaches of Margaret River only, a 60 km riverine system in Western Australia (Fig. 1). Reduction of their range within this river system has been attributed to the introduction of the smooth marron *Cherax cainii* (Decapoda: Parastacidae) in the 1980s [10]. The introduction of *C*. *cainii* to the lower reaches of Margaret River resulted in complete displacement of *C*. *tenuimanus* from the lower reaches within 13 years [10,11]. *Cherax cainii* has since spread up river, where it is now found in sympatry with all remaining *C*. *tenuimanus* populations [10,12].

Historically, marron was considered a single species, *C*. *tenuimanus*, following description of the species by Smith [9]. However, following the detection of significant genetic differences at four allozyme loci, as well as distinct morphological differences, two species were formally recognized as separate taxa, with the Margaret River marron retaining the original taxonomic name *C*. *tenuimanus* and the widespread smooth marron named *C*. *cainii* [11]. *Cherax tenuimanus* have setae on the cephalothorax that are completely absent in *C*. *cainii*, hence the common name of hairy marron. They are also characterized by a median carina that extends evenly and continuously to the cervical groove. In *C*. *cainii*, the median carina is more pronounced and either does not reach the cervical groove, or if it does, it is not continuous [11].

Evidence of hybridization between *C*. *tenuimanus* and *C*. *cainii* has been reported in several studies. An allozyme study by Austin and Ryan [11] found evidence of three distinct groups in the lower reaches of the Margaret River. The first group was morphologically typical of Margaret River (*C*. *tenuimanus*) marron. The second group was morphologically typical of marron from outside the Margaret River (*C*. *cainii*). Individuals in the third group, designated as hybrids, were morphologically intermediate and were heterozygous at three peptidase loci, which were fixed for alternate alleles in the other two groups. Austin and Ryan [11] further argued that a deficiency in heterozygosity at the peptidase loci indicated there were reproductive barriers between the two sympatric populations, suggesting they were good biological species. Subsequent studies using allozymes and morphology [13] and microsatellites [14] have also found evidence of interbreeding between two distinct morphotypes in the Margaret River. Finally, crossbreeding experiments conducted at the Department of Fisheries Pemberton Freshwater Research Centre have produced fertile female hybrids following matings between female *C*. *tenuimanus* and male *C*. *cainii* [15].

Identifying the population structure and the extent of introgression in Margaret River marron populations is critical for designing future conservation efforts to preserve the critically endangered *C*. *tenuimanus*. The present study uses recently developed microsatellite markers [16] to assess population structure and the extent of genetic mixing in a marron population from the upper reaches of the Margaret River. It was hypothesized that there would be two genetically distinct populations within the Margaret River, one representing *C*. *tenuimanus* and the other *C*. *cainii*, as well as a proportion of hybrid and backcrossed individuals. An investigation of the genetic variation within a captive breeding population of *C*. *tenuimanus* (initiated by the Western Australian Department of Fisheries) was also undertaken to validate the identity of the breeding individuals, in the anticipation that all individuals would be *C*. *tenuimanus*. Finally, we conducted simulations to test whether the observed levels of hybridization and backcrossing in the admixed Margaret River population were consistent with random mating between the two species.

## Methods

### Sample collection

One hundred and fifteen marron were captured from Boomerang Pool (33.87739°S, 115.29473°E) in the upper reaches of Margaret River in September 2012, using black box traps baited with chicken pellets, set 20 m apart along a transect. The traps were left overnight and checked in the morning for marron. A single leg was removed from each animal using forceps through atomizing (whereby the animal naturally drops their leg). Tissue samples were immediately stored in 100% ethanol. In addition to samples from the Margaret River, tissue samples were collected from 20 individuals from both Shannon River (34.76345°S, 116.37501°E) and Warren River (34.50561°S, 115.93629°E). Both the Shannon and Warren Rivers contain *C*. *cainii* populations only [11,17] and are at least 150 km from the Margaret River catchment boundary. Population samples from the Shannon and Warren Rivers were collected in 2012.

To provide reference material for *C*. *tenuimanus*, tissue samples from eight *C*. *tenuimanus* individuals (hereafter referred to as the *C*. *tenuimanus* control population) previously collected from Margaret River [16] as well as 42 individuals from a *C*. *tenuimanus* captive breeding population were included in the study. These marron were originally collected from the upper reaches of the Margaret River and were selected on the basis of their morphological traits.

All samples collected for our study were made by staff from the Western Australian Department of Fisheries. The collections were authorized by the Western Australian Department of Fisheries who have legislative responsibility for aquatic organisms. No specific permissions were required.

### DNA extraction, PCR and microsatellite genotyping

Genomic DNA was extracted from tissue samples using either a QIAGEN DNeasy Blood and Tissue Kit (www.qiagen.com) or a Xytogen animal DNA quick extraction kit (www.xytogen.com), following the manufacturer’s instructions. Genotypes at 13 microsatellite loci (*Cher01*, *05–08*, *10*, *14–15*, *19–21*, *24*, *26*) were determined for each individual using primers and polymerase chain reaction (PCR) conditions as described in Kennington et al. [16]. PCR products were analyzed on an ABI3700 sequencer using a GeneScan-500 LIZ internal size standard. Genotypes were scored using GENEMARKER version 1.9 (SoftGenetics, State College, PA, USA) software. The microsatellite data collected in this study has been deposited on the DRYAD website (data identifier: doi:10.5061/dryad.h00mt).

### Data analysis

The presence of null alleles was tested for each locus using the MICROCHECKER software package [18]. Levels of genetic variation in each population sample were quantified by calculating allelic richness (a measure of the number of alleles independent of sample size) and gene diversity. Tests for a deficit or excess in heterozygotes were carried out for each locus within each population using randomization tests, and the results were characterized using the *F*_IS_ statistic [19]. *F*_IS_ values that were significantly positive indicate a deficit of heterozygotes relative to random mating, and negative values indicate an excess of heterozygotes. Genotypic disequilibrium (GD) between each pair of loci was assessed by testing the significance of association between genotypes. Genetic differentiation between samples was assessed by calculating Weir and Cockerham’s [20] estimator of *F*_ST_. Estimates of *F*_IS_, *F*_ST_, tests for deficits in heterozygotes and genotypic disequilibrium were calculated using the FSTAT software package [21]. Differences in estimates of genetic variation and *F*_IS_ values among samples were tested using Wilcoxon’s signed-rank tests or Friedman’s ANOVAs (Analysis of Variance) with samples paired by locus. These analyses were performed using the statistical software package R (version 2.15.3).

In addition to pairwise *F*_ST_ values, population structure was assessed using the Bayesian clustering method of Pritchard et al. [22] and Falush et al. [23] implemented with the program STRUCTURE 2.1. This method identifies genetically distinct clusters (*K*) based on allele frequencies across loci. All analyses were based on an ancestry model that assumed admixture and correlated allele frequencies. No prior information about the origin of the samples was used. Ten independent runs were performed for each value of *K* (1–10) with a burn-in of 10,000 followed by 100,000 Markov Chain Monte Carlo (MCMC) iterations. The most likely number of clusters was assessed by comparing the likelihood of the data for different values of *K* and using the *ΔK* method of Evanno et al. [24]. We also used the program NEWHYBRIDS [25] to determine the ancestry of all individuals from the Margaret River population. This program also uses a MCMC procedure to assign individuals to different generational classes: pure species 1 or 2, F_1_ or F_2_ hybrids and F_1_ backcrosses to each parent. Analyses were run using Jeffreys priors with 500,000 sweeps and a 50,000 burn-in value. No prior allele frequency information was used in the analysis. We used a posterior probability value of 0.50 as a threshold value for assignment of an individual to a generational class.

### Genetic introgression and the decay of genotypic disequilibrium and population structure under random mating

To determine whether the observed levels of hybridization and backcrossing in the admixed Margaret River population are consistent with random mating and whether there has been sufficient time for random mating to breakdown GD and genetic differentiation between subpopulations, simulated data sets were created using a modified version of EASYPOP 2.0.1 [26]. The simulations were based on a simple model consisting of two diploid subpopulations with complete mixing (random mating) between them. The total number of individuals (i.e. both subpopulations combined) was set at 10,000 with an equal sex ratio. However, the number of individuals in each subpopulation varied to reflect the different proportions of *C*. *cainii* and *C*. *tenuimanus* in the upper reaches of the Margaret River since 2002 [0.7 and 0.3 respectively, 10], when the last genetic assessment of the population was made. The mutation rate was set at 0.001 [27,28]. Both single step and two-phased models of mutation were used. However, because both mutation models gave qualitatively similar results, only the results with the single step mutation model are presented.

The simulations ran for three generations (the estimated number of generations since 2002 assuming a generation time of three years [29]) and were based on 12 loci with free recombination and a maximum of seven alleles per locus (the average number of alleles per locus in the Margaret River population samples). Simulations were started with either maximum or intermediate (*F*_ST_ ~ 0.5) allele frequency differences between subpopulations. In models with maximum genetic divergence, subpopulations were fixed for different alleles at each locus. Subpopulations with intermediate genetic divergences were set up by randomly selecting alleles from all possible allelic states for one subpopulation and randomly selecting alleles from a subset of possible allelic states for the other subpopulation. This also resulted in a difference in allelic richness between subpopulations, which is what we observed in the *C*. *tenuimanus* samples.

At each generation 40 individuals were randomly selected from each subpopulation (80 individuals in total) and used to test for population structure and ancestry using STRUCTURE and NEWHYBRIDS, as undertaken with the original data. Ten simulated data sets were generated and tested for genetic differentiation for each model. Simulated data sets from each generation were also analyzed with FSTAT to assess levels of GD between pairs of loci. The significance of differences between the observed and simulated results was tested using one sample *t*-tests.

## Results

### Genetic variation

One locus (*Cher08*) was identified as having null alleles in the control Shannon River population using MICROCHECKER, and was therefore removed from further analyses for all populations.

Genetic diversity statistics for the remaining 12 loci were typically higher in the Margaret River population sample than the *Cherax cainii* populations from Shannon and Warren River and the *C*. *tenuimanus* control and captive breeding populations (Table 1). Friedman’s ANOVA indicated significant differences in allelic richness and gene diversity across population samples (*P* < 0.001 in all cases). Further pairwise testing using Wilcoxon’s signed-rank tests revealed significantly higher allelic richness and gene diversity within the population sample from Margaret River compared to all other population samples (*P* ≤ 0.001 in all cases). There was also a significant difference in gene diversity between the *C*. *tenuimanus* control and *C*. *cainii* Warren River population samples (*P* = 0.045), with higher gene diversity in the *C*. *cainii* sample. All other pairwise tests were non-significant.

**Table 1:** Genetic variation and genotypic disequilibrium (GD) within each population sample.

Site	*N*	*A*_R_	*H*	*F*_IS_	*GD*
*C*. *cainii*					
Shannon River (SR)	20.0 (0.0)	2.0 (0.3)	0.26 (0.09)	0.11	0
Warren River (WR)	20.0 (0.0)	2.7 (0.5)	0.35 (0.09)	-0.03	0
*C*. *tenuimanus*					
Controls (CON)	8.0 (0.0)	1.6 (0.2)	0.10 (0.04)	-0.10	0
Captive breeding (CB)	42.0 (0.0)	2.4 (0.3)	0.21 (0.06)	0.19*	14
CB—hybrids removed	38.0 (0.0)	1.8 (0.3)	0.14 (0.06)	0.11	0
Admixed					
Margaret River (MR)	114.3 (0.4)	4.3 (0.5)	0.67 (0.04)	0.32*	66

*N* is the mean sample size per locus, *A*_R_ is allelic richness, *H* is gene diversity and *F*_IS_ is the inbreeding coefficient. Standard errors are in parentheses. *F*_IS_ estimates significantly greater than zero after correction for multiple comparisons are denoted with an asterisk.

Population samples from Margaret River and the *C*. *tenuimanus* captive breeding population had significantly positive multilocus *F*_IS_ values indicating a higher than expected proportion of homozygotes (Table 1). There was also genotypic disequilibrium (GD) in these population samples. GD was highest in the Margaret River population samples with 66 pairs of loci in significant GD. The *C*. *tenuimanus* captive breeding population had 14 pairs of loci in GD when hybrids were included in the analysis. However, the removal of hybrid individuals resulted in no pairs of loci being in GD and the *F*_IS_ value became non-significantly different to zero (Table 1).

### Population structure and genetic introgression

Significant differences in allele frequencies were evident between all population samples except between the *C*. *tenuimanus* control and captive breeding populations (Table 2). Pairwise *F*_ST_ values indicate that the largest differences in allele frequency were between the *C*. *tenuimanus* and the *C*. *cainii* population samples. Allele frequencies in the Margaret River population sample were most similar to the Shannon and Warren River *C*. *cainii* populations. Pairwise *F*_ST_ values between the Margaret River and *C*. *cainii* population samples ranged from 0.20 to 0.26 compared to 0.31 to 0.35 between the Margaret River and *C*. *tenuimanus* population samples.

**Table 2:** Pairwise *F*_ST_ estimates between population samples.

	SR	WR	CON	CB	MR
*C*. *cainii* (SR)	–				
*C*. *cainii* (WR)	0.46*	–			
*C*. *tenuimanus* (Con)	0.77*	0.73*	–		
*C*. *tenuimanus* (CB)	0.75*	0.73*	0.00	–	
Margaret River (MR)	0.26*	0.20*	0.34*	0.35*	–

*F*_ST_ estimates significantly greater than zero after correction for multiple comparisons are denoted with an asterisk.

**Fig 2:**
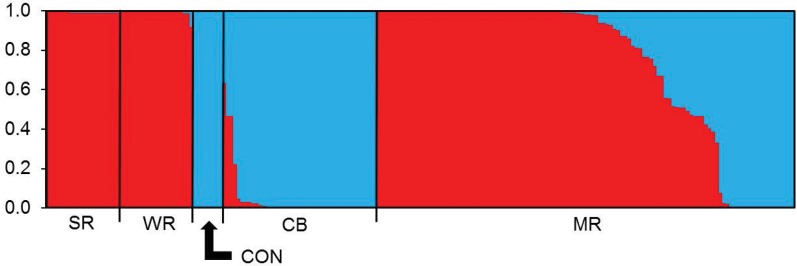
Summary of the clustering results assuming two admixed populations (*K* = 2). Each individual is represented by a bar showing the individual’s estimated membership to a particular cluster (represented by different colours). Black lines separate samples from different populations. Abbreviated population names are defined in Table 1.

Bayesian clustering analysis indicated *K* = 2 as the most likely number of clusters according to plots of L(*K*) and Δ*K* across different values of *K*. These clusters were representative of *C*. *cainii* and *C*. *tenuimanus*, as determined by the assignment of all individuals from the *C*. *tenuimanus* control population sample to one cluster and all individuals from the *C*. *cainii* Warren and Shannon River populations to the other genetic cluster (Fig. 2). Individuals from the *C*. *tenuimanus* captive breeding population sample were predominantly assigned to the cluster representing *C*. *tenuimanus*, though a few individuals were a mixture of both clusters and appeared to be hybrids or backcrosses with *C*. *cainii*.

Individuals in the Margaret River population samples were assigned to one genetic cluster or were a mixture of both. Using a threshold value of ≥90% membership to a single cluster to be considered a pure *C*. *tenuimanus* or *C*. *cainii*, the Margaret River population sample was calculated to consist of 58.3% *C*. *cainii*, 18.3% *C*. *tenuimanus* and 23.5% hybrids. The NEWHYBRIDS analysis of this population sample indicated that most hybrids were F_1_ or F_2_ generations with lower frequencies of backcrosses to each parental species. The analysis identified 60.5% *C*. *cainii*, 18.4% *C*. *tenuimanus*, 5.3% F_1_, 10.5% F_2_ and 5.3% backcross to *C*. *cainii*. There were no individuals identified as being a backcross to *C*. *tenuimanus*.

### Genetic introgression and the decay of genotypic disequilibrium and population structure under random mating

Our simulations suggest that genetic introgression should occur rapidly under random mating. When simulations were started with maximum genetic divergence (i.e. subpopulations were fixed for different alleles at each locus) the percentage of individuals that were assigned to the parental subpopulations (i.e. not admixed) after three generations of random mating was much lower than observed, while the percentage of individuals that were F_2_ or backcrossed was much higher than observed (Table 3). Similar patterns were observed when simulations were started with intermediate allele frequency differences between subpopulations (*F*_ST_ ~ 0.5), though the differences between observed and simulated results were greater (Table 3).

**Table 3:** Summary of the simulation results.

		Intermediate genetic divergence			Maximum genetic divergence		
Analysis	Observed	Mean	*t*	*P*	Mean	*t*	*P*
*NEWHYBRIDS*							
Parental (%)	78.9	4.6 (0.8)	–95.4	<0.001	23.7 (1.5)	–36.9	<0.001
F_1_ (%)	5.3	2.7 (1.3)	–2.0	0.074	9.6 (0.7)	6.4	<0.001
F_2_ (%)	10.5	28.5 (3.2)	5.6	<0.001	14.7 (1.6)	2.7	0.023
Backcross (%)	5.3	64.2 (4.2)	23.5	<0.001	51.9 (5.0)	20.5	<0.001
*FSTAT*							
Number of loci in GD	66.0	1.0 (0.4)	–145.3	<0.001	39.9 (4.2)	–6.2	<0.001
*STRUCTURE*							
Number of genetic clusters (*K*)	2.0	2.3 (0.2)	0.0	1.000	2.0 (0.0)	0.0	1.000

Parameter values are means from the analysis of 10 simulated data sets generated using a model with three generations of random mating between subpopulations that had intermediate (*F*_ST_ ~ 0.5) or maximum allele frequency differences between them. Standard errors are in parentheses.The significance of differences between the observed and simulated results were tested using a one sample *t*-test.

Stronger agreement between the simulated and observed data sets was found when assessing population structure. As found in the actual data, Bayesian clustering analysis of the simulated data sets using STRUCTURE predominantly indicated *K* = 2 as the most likely number of clusters, irrespective of whether the simulations were started with maximum and intermediate genetic divergence between subpopulations. The simulations also indicated that high levels of genotypic disequilibrium (GD) are not unexpected after three generations of random mating under some scenarios. However, the observed levels of GD were significantly higher than expected under random mating (Table 3).

## Discussion

The Margaret River population of marron is admixed, containing individuals that are pure *C*. *tenuimanus*, pure *C*. *cainii*, as well as hybrids and backcrossed individuals. There are approximately three times more *C*. *cainii* present at Boomerang Pool than *C*. *tenuimanus* or hybrids, which are present in approximately equal proportions. De Graaf et al. [10] reported that since 2002 the proportion of pure *C*. *tenuimanus* in the upper reaches of the Margaret River was approximately 30% (a decline from 100% in 1995), and had remained stable until their study in 2009. Microsatellite analysis indicates that the proportion of *C*. *tenuimanus* in the site sampled is less than 20% of the total marron population. It is unclear whether this difference reflects a temporal change in the composition of the population in the upper reaches of the Margaret River or is due to the microsatellites being better able to detect hybrid individuals, thereby reducing the reported proportion of pure *C*. *tenuimanus*. This study also used a different sampling site to previous studies (precise sampling locations in previous studies were not given), which may also explain the different estimates.

The identification of an admixed Margaret River population validates the results of previous allozyme [11], allozyme and morphological [13], and microsatellite [14] studies detecting interbreeding between C. *tenuimanus* and *C*. *cainii*. Previous estimates of the level of hybridization in the upper reaches of the Margaret River range from 7.5% in 1981 to 12.5% in 1992 [11]. The NEWHYBRIDS analysis in this study indicates that 21.1% of the population sampled had hybrid or backcrossed origin. This represents a 1.9-fold increase in the proportion of hybrids to previous estimates, and in conjunction with the apparent decline of pure *C*. *tenuimanus*, it appears that hybridization and introgression still pose a serious threat to the viability of the current *C*. *tenuimanus* population.

The introduction of non-indigenous crayfish species is regarded one of the greatest threats to freshwater crayfish biodiversity [6]. For example, populations of *Orconectes propinquus* and *O*. *virilis* in North America have been completely replaced by their congener *O*. *rusticus* [6,30]. While ecological mechanisms seem to be the only factors driving the extirpation of *O*. *virilis* by *O*. *rusticus*, hybridization and introgression appear to play an important role in the extirpation of *O*. *propinquus* [6]. Indeed, it has been estimated that genetic introgression increased the spread of *O*. *rusticus* genes by 36% above ecological replacement mechanisms alone [30]. This process also appears to be occurring for *C*. *tenuimanus* populations, with their complete replacement by *C*. *cainii* in the lower and middle reaches of Margaret River within 13 years of its introduction to this river system [10,11]. This study shows that in addition to ecological displacement, there is considerable introgression between the two species, placing further pressure on the declining *C*. *tenuimanus* populations.

It is not known why *C*. *cainii* are displacing *C*. *tenuimanus* from their home range. The rate of displacement of *C*. *tenuimanus* in the lower and middle reaches suggests competitive mechanisms are in place [11]. There is some suggestion of a difference in the timing of reproduction for each species based on females capture rates [12], however, this requires more research to be conclusive. Should it prove to be the case, earlier breeding times in *C*. *cainii* may give rise to larger *C*. *cainii* males, which may prevent *C*. *tenuimanus* males getting access to mates, contributing to the decline of the pure *C*. *tenuimanus* population. Another hypothesis is that earlier breeding times give *C*. *cainii* offspring a competitive size advantage over *C*. *tenuimanus* offspring, making them less susceptible to predation or better able to secure limited resources (e.g. suitable habitats). Sympatric juvenile coho salmon (*Oncorhynchus kisutch*) and steelhead trout (*O*. *mykiss*) experience strong asymmetric competition, whereby coho salmon are naturally larger than steelhead trout resulting in poorer habitat selection by the smaller trout [31]. Pseudocompetition may also be occurring indirectly due to the general decline of the *C*. *tenuimanus* population, resulting in fewer conspecific encounters and matings between *C*. *tenuimanus* individuals. Investigating these processes in an experimental setting would provide valuable insights into the factors causing the decline of *C*. *tenuimanus* populations and increasing proportion of *C*. *cainii* and hybrids in Margaret River.

The Margaret River admixed population exhibits significantly higher levels of gene diversity and allelic richness when compared to populations of pure *C*. *tenuimanus* and *C*. *cainii*. Such patterns are not unexpected in recently admixed populations [32,33]. The Margaret River population sample also had a significantly positive inbreeding coefficient, a sign of higher than expected homozygosity. Positive inbreeding coefficients may reflect non-random mating between the two species or selection against hybrid individuals. Alternatively, it may be that migration of *C*. *cainii* from the lower reaches of the Margaret River is preventing the population in the upper reaches reaching equilibrium, though the relatively stable proportions of *C*. *cainii* in the upper reaches over the last 11 years suggests that migration rates are low.

The pairwise *F*_ST_ values revealed there were significant differences in allelic frequencies between the Margaret River population sample and all others. Further analyses using only individuals identified as pure *C*. *cainii* based on the STRUCTURE results may shed light on the origins of *C*. *cainii* introduced to the Margaret River. However, it would first be necessary to sample widely throughout the *C*. *cainii* range to confidently address this question. The significant differences in allelic frequencies between the *C*. *cainii* Shannon River and Warren River population samples suggest that the population divergences required for such a study are present. This result is also in agreement with the only previous microsatellite study [14], which identified significant genetic divergences among populations of marron sampled from different river systems.

### Taxonomic and management implications

The taxonomic status of *C*. *tenuimanus* and *C*. *cainii* as independent species has been questioned due to reports of widespread hybridization in natural and artificial environments [10]. While interbreeding between the two species was confirmed in this study, our simulations suggest that the levels of introgression are much lower than would be expected under random mating. This suggests there are partial reproductive barriers between *C*. *tenuimanus* and *C*. *cainii*. Further, the analyses clearly identified the presence of two distinct genetic populations, corresponding to each of the current species. Therefore, regardless of interbreeding, *C*. *tenuimanus* and *C*. *cainii* should continue to be considered as separate evolutionary significant units [8] and distinct species on the basis that they form separate genotypic and phenotypic clusters [34].

Although lower than expected under random mating, hybridization and introgression with *C*. *cainii* should be regarded as serious threats to *C*. *tenuimanus*. Without intervention, demographic and genomic extinction of *C*. *tenuimanus* in the wild appears inevitable. More accurate methods to rapidly identify *C*. *cainii* and hybrid individuals are being developed to assist with projects aimed at removing them from the upper reaches of the Margaret River. In addition, intensive sampling of the marron in the upper reaches is being used to determine the levels of effort required for physical removal to achieve significant benefits for the *C*. *tenuimanus* population. There is also an effective captive breeding program underway which, in light of these results, remains a high priority. Although the *C*. *tenuimanus* control population had lower gene diversity than the *C*. *cainii* Warren River population, allelic richness and gene diversity in the *C*. *tenuimanus* captive breeding population were comparable to levels in both the Warren and Shannon River populations of *C*. *cainii*, which will be important for the long-term viability of the species. Regular monitoring is needed to ensure that genetic diversity is maintained in the captive breeding population. Furthermore, to maintain the genetic integrity of the captive population, all wild-caught individuals added to the captive breeding population should be subjected to microsatellite genotyping to ensure that hybrid or backcrossed individuals are not mistakenly introduced.

## References

[1] Levin DA. Hybridisation and extinction: In protecting rare species, conservationists should consider the dangers of interbreeding, which compound the more well-known threats the wildlife. Am Sci. 2002;90: 254–261.

[2] Mack RN, Simberloff D, Lonsdale M, Evans H, Clout M, Bazzazz FA. Biotic invasions: causes, epidemiology, global consequences, and control. Ecol Appl. 2000;10: 689–710.

[3] Mooney HA, Cleland EE. The evolutionary impact of invasive species. P Natl Acad Sci USA. 2001;98: 5446–5451. 11344292 10.1073/pnas.091093398 PMC33232

[4] Hardig TM, Allison JR, Schilling EE. Molecular evidence of hybridization between *Liatris oligocephala* (Asteraceae) and more-widespread congener: a preliminary assessment of the potential for extinction. Castanea. 2005;70: 246–254.

[5] Homyack A, Vashon JH, Libby C, Lindquist EL, Locj S, McAlpine DF, et al Canada lynx-bobcat (*Lynx canadensis* x *L*. *rufus*) hybrids at the southern periphery of *Lynx* range in Maine, Minnesota and New Brunswick. Am Midl Nat. 2008;159: 504–508.

[6] Perry WL, Lodge DM, Feder JL. Implications of hybridisation between introduced and resident *Orconectes* crayfishes. Conserv Biol. 2001;15: 1656–1666.

[7] Rhymer JM, Simberloff D. Extinction by hybridisation and introgression. Annu Rev Ecol Syst. 1996;27: 83–109.

[8] Allendorf FW, Luikhart G. Conservation and the genetics of populations. Carlton, Australia: Blackwell Publishing; 2009.

[9] Smith G. The freshwater crayfishes of Australia. P Zool Soc Lond. 1912;10: 144–170.

[10] De Graaf M, Lawrence C, Vercoe P. Rapid replacement of the Critically Endangered hairy marron by the introduced smooth marron (Decapoda, Parasatcidae) in the Margaret River (Western Australia). Crustaceana. 2009;82: 1469–1476.

[11] Austin CM, Ryan SG. Allozyme evidence for a new species of freshwater crayfish of the genus *Cherax* Erichson (Decapoda: Parastacidae) from the south-west of Western Australia. Invertebr Syst. 2002;16: 357–367.

[12] Bunn JJS. Investigation of the replacement of Margaret River hairy marron *Cherax tenuimanus* (Smith) by smooth marron *C* *cainii* Austin. Perth, Western Australia: Edith Cowan University 2004.

[13] Bunn JJS, Koenders A, Austin CM, Horwitz P. Identification of hairy, smooth and hybrid marron (Decapoda: Parastacidae) in the Margaret River: morphology and allozymes Proceedings of International Symposium on Freshwater Crayfish. Gold Coast, Australia International Association of Astacology; 2008 pp. 113–121.

[14] Imgrund JA. Population genetic analysis of the freshwater crayfish, *Cherax tenuimanus*: Curtin University of Technology. 1998. 10.1002/elps.1150180932 9378141

[15] Lawrence C. Improved performance of marron using genetic and pond management strategies. Final report to Fisheries Research nad Development Corporation (Project No. 2000/215). Fisheries Research Contract Report. 2007;17: 40–49.

[16] Kennington WJ, Guildea C, Lukehurst SS, Hitchen Y, Gardner MG, Duffy R, et al Isolation and characterization of 13 polymorphic microsatellite loci for the smooth *Cherax cainii* and hairy marron *C*. *tenuimanus* (Decapoda: Parastacidae). Conserv Genet Resour. 2014;6: 337–339.

[17] Austin CM, Knott B. Systematics of the freshwater crayfish genus *Cherax* Erichson (Decapoda: Parastacidae) in southeastern Australia: electrophoretic, morphological and habitat variation. Aust J Zool. 1996;44: 223–258.

[18] van Oosterhout C, Hutchinson WF, Wills DPM, Shipley P. MICROCHECKER: software for identifying and correcting genotyping errors in microsatellite data. Mol Ecol Notes. 2004;4: 535–538.

[19] Wright S. The genetical structure of populations. Ann Eugen. 1951;15: 323–354. 24540312 10.1111/j.1469-1809.1949.tb02451.x

[20] Weir BS, Cockerham CC. Estimating *F*-statistics for the analysis of population structure. Evolution. 1984;38: 1358–1370. 28563791 10.1111/j.1558-5646.1984.tb05657.x

[21] Goudet J. FSTAT, a program to estimate and test gene diversities and fixation indices (version 2.9.3); 2002. Available: http://www.unil.ch/izea/softwares/fstat.html.

[22] Pritchard JK, Stephens M, Donnelly P. Inference of population structure using multilocus genotype data. Genetics. 2000;155: 945–959. 10835412 10.1093/genetics/155.2.945 PMC1461096

[23] Falush D, Stephens M, Pritchard JK. Inference of population structure using multilocus genotype data: linked loci and correlated allele frequencies. Genetics. 2003;164: 1567–1587. 12930761 10.1093/genetics/164.4.1567 PMC1462648

[24] Evanno G, Regnaut S, Goudet J. Detecting the number of clusters of individuals using the software STRUCTURE: a simulation study. Mol Ecol. 2005;14: 2611–2620. 15969739 10.1111/j.1365-294X.2005.02553.x

[25] Anderson EC, Thompson EA. A model-based method for identifying species hybrids using multilocus genetic data. Genetics. 2002;160: 1217–1229. 11901135 10.1093/genetics/160.3.1217 PMC1462008

[26] Balloux F. EASYPOP (version 1.7): a computer program for the simulation of population genetics. J Hered. 2001;92: 301–302. 11447253 10.1093/jhered/92.3.301

[27] Ellegren H. Microsatellite mutations in the germline: implications for evolutionary inference. Trends Genet. 2000;16: 551–558. 11102705 10.1016/s0168-9525(00)02139-9

[28] Goudet J, Perrin N, Waser P. Tests for sex-biased dispersal using bi-parentally inherited genetic markers. Mol Ecol. 2002;11: 1103–1114. 12030985 10.1046/j.1365-294x.2002.01496.x

[29] Morrissy NM. Spawning variation and its relationship to growth rate and density in the marron, *Cherax tenuimanus* (Smith). Fisheries Res Bull Western Australia. 1975;16: 1–32.

[30] Perry WL, Lodge DM, Feder JL. Importance of hybridisation between indigenous and non-indigenous freshwater species: an overlooked threat the North American biodiversity. Syst Biol. 2002;51: 255–275. 12028732 10.1080/10635150252899761

[31] Young KA. Asymmetric competition, habitat selection, and niche overlap in juvenile salmonids. Ecology. 2004;85: 134–149.

[32] Binks RM, Kennington WJ, Johnson MS. Rapid evolutionary responses in a translocated population of intertidal snail (*Bembicium vittatum*) utilise variation from different source populations. Conserv Genet. 2007;8: 421–1429.

[33] Kennington WJ, Hevroy TH, Johnson MS. Long-term genetic monitoring reveals contrasting changes in the genetic composition of newly established populations of the intertidal snail *Bembicium vittatum* . Mol Ecol. 2012;21: 3489–3500. doi: 10.1111/j.1365-294X.2012.05636.x 22612539 10.1111/j.1365-294X.2012.05636.x

[34] Hausdorf B. Progress toward a general species concept. Evolution. 2011;65: 923–931. doi: 10.1111/j.1558-5646.2011.01231.x 21463293 10.1111/j.1558-5646.2011.01231.x

